# The Rational Therapeutics of Pyorrhœa Alveolaris

**Published:** 1895-08

**Authors:** Henry H. Burchard

**Affiliations:** Philadelphia


### THE RATIONAL THERAPEUTICS OF PYORRHŒA ALVEOLARIS.

BY HENRY H. BURCHARD, M.D., D.D.S., PHILADELPHIA.

In no other instance, except it may be the vomiting of pregnancy, is the truth of the maxim—"in a multitude of remedies there is no remedy"—better illustrated than in the therapeutics of the condition known as pyorrhœa alveolaris. There are but few drugs of the classes of escharotics, antiseptics, and astringents which have not been applied. Each one has had its advocates, subscribers to its efficiency; and just as great a number have declared the same agent worthless. Many of the drugs have been described as almost specific in their action. Undoubtedly a specific is what is wanted, but the first step in finding a specific remedy is to ascertain that the disease itself is specific.

As pyorrhœa is demonstrably significant of, it may be, a dozen or more disease conditions, one specific will scarcely cover the field for all.

In any disease the problem confronting a therapist is a dual one: removing cause and remedying effect. It by no means follows that the removal of a cause is followed by the disappearance of all effects. The phenomena represented by this secondary element may be the important ones.

Nearly all critics of the literature of this subject comment upon the lack of rationality evinced in the therapeutics of the disorder.

There is no reason why this dental condition should be placed beyond kinship with all other diseased states. Commonly it is viewed as being in dental disorders what carcinoma is in surgical or tuberculosis in medical. The classification is unwarranted. The use of the term, applied as it is, is most irrational; it is merely the description of a symptom, not of a disease. To say that a patient has pyorrhœa alveolaris is less definite than to say he or she has an ulcer; for the latter is something tangible, the former but a statement that a patient has pus oozing from a tooth socket.

Dr. M. L. Rhein has presented in the *Dental Cosmos* a list of disease conditions, in all of which this symptom may form an attendant.

In the matter of nomenclature, for the virulent variety Dr. G. V. Black has given us one of the most descriptive and happily applicable terms in that of phagedenic pericementitis. This is frequently applied as a synonym of pyorrhœa alveolaris, a contradiction of Dr. Black's written description. As he describes phagedenic pericementitis, it is a disease process, distinct in itself, of which pyorrhœa is but one symptom, and may not be even that. The present writer maintains that this title, given by Dr. Black, is one of the best in all dental nomenclature, and should be retained until such time as it may be fitly superseded by an absolute title found upon the undisputed cause of the disease states.

The name pyorrhœa alveolaris is deliberately chosen as a heading to this essay, so that phagedenic pericementitis may be included.

As before stated, the therapeutic problem confronting us is that of the removal of causes and the cure of effects.

Irrespective of the predisposing causes and those exciting causes which gave origin to the present diseased condition, the disorder known as pyorrhœa has resulting from or accompanying it a tolerably distinct set of surgical conditions to be overcome. As a rule they are as follows: Detachment of the gum from the cervices of the teeth; the gums themselves have any stage of localized inflammation, usually a tumefaction; a chronic congestion; pockets are formed between the teeth and gums, and in the pockets are firmly adherent deposits; an increasing loss of pericementum, and it may be of cementum also, with a molecular destruction of the alveolar walls. In consequence of this progressive loss of retentive apparatus the teeth loosen and the looseness is increased by the strain of mastication, which tends to force the organs from their scant support. The same force, by increasing the arc of movement at the apex of the root, may cause amputation of the vascular and neural supply to the pulp.

Many of these teeth which are the victims of persistent pyorrhœa have the continuation of the pus formation due to the presence of the dead and decomposing pulp, which has become the breeding-ground of micro-organisms. So it may be well to mention here that the opening and thorough sterilizing of the pulpchamber may be required as one therapeutic step. Such a procedure has determined a cure in some instances.

It will be seen there is a set of conditions; each one is a definite indication for treatment. Rational therapeutics demands that each item shall receive intelligently-directed consideration.

The ultimate object being the healthy retention of the teeth, the elements which threaten their retention should be eliminated in the order of their immediate power. Undue strain from mastication is the first factor, so that faults of occlusion should be first corrected. This is accomplished through a judicious trimming of the teeth. As this operation might, in itself, represent sufficient force to dislodge the teeth, while the grinding is being done, a temporary splint should be applied,—ligatures suffice for this purpose.

The teeth are now in the condition of subluxation. A fixed splint is therefore required to keep them at surgical rest. The operator's ingenuity will suggest an applicable form for this,—rings, ligatures, plate, or some one, or a modification, of Dr. Jackson's devices.

The writer uses more often than any other means a thin plate of swaged metal, fitted to the teeth, and several adjoining ones, and cemented with oxyphosphate of zinc.

One surgical indication has been met. The greatest source of irritation remaining is foreign bodies; these being represented by the calcic deposits. The greater (at least the grosser) part of these is removed while the temporary splint is in use. *It is absolutely essential that the last vestige of these deposits be removed to furnish reasonable hope of cure.*

General experience indicates that solvents must play a secondary part to instrumental means for the attainment of the object sought. In this connection, trichloracetic acid will check oozing and permit a better view of the pockets, and to some extent soften the deposits. Aromatic sulphuric acid destroys carious bone, serves as a stimulant and antiseptic, but as a solvent of calculi it plays a small part. These two strong acid applications are mentioned to emphasize the importance of thorough instrumentation for the desired end.

Presuming now the immobility of the teeth secured, and this with a normal articulation less the thickness of the splint, every particle of foreign material removed from the roots, and there still remain morbid conditions. (It may be observed, parenthetically, that in some cases these deposits are so strongly adherent as to require the use of a file for their removal, even after extraction.) The cementum of the tooth or teeth deprived, it may be, of a great extent of its nutrient membrane, a loss of bony walls of support, and the tissues about in a state of subacute inflammation. Blind pockets present, containing pus, organisms, and broken down tissue, and open to the access of pathogenic material.

The organisms must be first destroyed and the dead tissues removed. Considering the tortuosity of the recesses to be acted upon, it is evident that strong solutions of hydrogen peroxide are the indication. The open pockets are now the menace, as they permit the access of micro-organisms, so the indication is their closure, the evident need being an annular elastic bandage. In addition to this the destruction of fresh crops of micro-organisms must be assured. A mechanical splint for the purpose named being impracticable, its substitute is found in astringents, which, by causing the tissues to contract, form the soft parts themselves into a bandage. The continued use of an antiseptic is essential.

For the combined purpose of astringent and antiseptic ablutions zinc chloride meets the need. As the nutritive change to be induced is a constructive, not a destructive one, this solution should be in strength fit as a stimulant, not a caustic, say one or two per cent. Its action is, however, inferior to zinc iodide as regards special stimulation.

Other surgical means for the induction of tissue growth in addition to stimulants, such as sponge grafts, offer but a faint hope, owing to the difficulties of perfect sterilization and the debasement of the tissues. The disease arrested, and whatever loss of alveolar process there may be is a permanent loss.

The possibilities of judicious replantation in selected cases are left for experimentation.

Single remedies of classes have been selected here for prescription, with a distinct purpose.

As far as local therapeusis is concerned, this is a statement of conditions and requirements, and it is more rational to use the means at our disposal (with discretion) than it is to multiply remedies, most of which are at present of doubtful value. The drugs now in use meet the needs as these needs are understood, so that labor in the lengthening of drug lists is poorly directed.

Our therapeutic sources are fully abreast or are in advance of our knowledge of the etiology and pathology involved in pyorrhœa; and if there is a general failure of therapeutic application, it is probably because there are disease elements of which we have not full cognizance, or that we have not removed all causes known.

It is conceded that some forms of pyorrhœa are of purely local significance; and these are undoubtedly curable and cured by local therapeusis of the type mentioned above. Failure in these cases is due to incompleteness in some step of the therapeutics, usually in the removal of deposits.

It is generally accepted that some forms of pyorrhœa have a constitutional significance. Some constitutional state acting as a predisposing and, it may be, existing cause; producing a condition either identical with, similar to, or differing in some particulars from the one described.

Evidently in these cases to effect a cure requires the correction of the constitutional cause, if it still be active. This proviso is made, because a constitutional cause may have passed over the anatomical part involved like a wave, and then the only evidence remaining of its action is the local disease. Cure is delayed, is hopeless so long as the general condition persists. Even if cured, the local trouble may return with any recurrence of the constitutional malady.

Any cause or causes known to give origin to this local condition must be eliminated in the order of their probability. As to the catarrhal aspect, a proper inquiry is, What is the cause of the catarrh? This itself may be the result of gout, a common cause of pericemental degeneration. Whatever the cause, it is certain that pyorrhœa, as any other condition, is curable only by removal of the cause and remedying the effects. This applies no matter what the cause. When we discover these, our therapeutic resources will no doubt be found sufficient, and the formidable drug lists will be relegated to their proper place,—"innocuous desuetude."

Personally I am of the opinion that next to incomplete removal of deposits as the greatest cause of failure in therapeusis is a lack of care in determining for each case the individual elements which are involved in the etiology, for, as to this matter, cases vary, and vary greatly.

I have seen so many cases of tooth-loss, attended by pyorrhœa alveolaris, due, and due alone, to malocclusion, that I am prompted to add this as causative of a distinct class. Incidental to this, this loss through malocclusion occurs in the plethoric as commonly as in the anæmic patient.

The local causes are quite sufficient to explain the disorder, and it rapidly subsides with the correction of the false occlusion. They in no way resemble the gouty cases, except, perhaps, in a common symptom,—pyorrhœa.

